# Maternal Identity and Role Balance in Pregnancy: Construction and Validation of the Maternal Role Integration Questionnaire (MRIQ-P)

**DOI:** 10.3390/bs16040578

**Published:** 2026-04-11

**Authors:** Alejandro García-Romero, Cecilia Peñacoba, Patricia Catalá

**Affiliations:** Department of Psychology, Rey Juan Carlos University, 28922 Alcorcón, Madrid, Spain; alejandro.garcia@urjc.es (A.G.-R.); cecilia.penacoba@urjc.es (C.P.)

**Keywords:** maternal identity, maternal role integration, pregnancy, perinatal psychology, role balance

## Abstract

Background: Pregnancy represents a major identity transition, yet most perinatal assessments focus primarily on emotional symptoms rather than on how women integrate the maternal role into their broader identity and life context. Difficulties in maternal role integration may constitute an early vulnerability factor for psychological distress. This study aimed to develop and validate the Maternal Role Integration Questionnaire—pregnancy version (MRIQ-P), a brief instrument designed to assess maternal identity and role balance during pregnancy, and to examine its clinical relevance for perinatal mental health. Methods: A sequential mixed-methods design was employed. Phase 1 involved focus groups with pregnant women (n = 17) and cognitive debriefing to generate and refine items. Phase 2 included expert evaluation of content validity. In Phase 3, the MRIQ-P was psychometrically validated in a sample of pregnant women (n = 256), randomly divided into exploratory (n = 83) and confirmatory (n = 173) subsamples. Exploratory and confirmatory factor analyses were conducted, along with reliability analyses, tests of convergent, discriminant, incremental, and measurement invariance validity. Results: Analyses supported a bifactor structure comprising a general factor of maternal role integration and two specific dimensions: Maternal Identity and Balance of the Maternal Role. The final 8-item version demonstrated excellent internal consistency for the total score (α = 0.96) and subscales (α = 0.98 for Maternal Identity and α = 0.98 for Balance of the Maternal Role), as well as measurement invariance across primiparous and multiparous women. Higher maternal role integration was associated with greater self-esteem, positive affect, and life satisfaction, and with lower anxiety, depression, prenatal distress, and maternal ambivalence. Importantly, MRIQ dimensions explained additional variance in antenatal depression and dispositional guilt beyond established psychological predictors, supporting its incremental and potential clinical utility. Conclusions: The MRIQ is a brief, psychometrically robust, and clinically relevant instrument for assessing maternal role integration during pregnancy. By capturing identity- and role-related processes that are not directly addressed by symptom-based screening tools, it may contribute to early identification of vulnerability and to more comprehensive perinatal psychological assessment in healthcare settings.

## 1. Introduction

Pregnancy constitutes one of the most significant identity transitions in women’s lives, involving biological, relational, and symbolic changes that require a profound reorganization of the self. Rubin’s classical theoretical work conceptualized motherhood as a process of maternal role attainment, emphasizing that pregnancy initiates a gradual psychological process through which women begin to internalize the maternal role and construct a maternal identity (Rubin, 1967, 1984). This identity-related work begins during gestation and continues in the postpartum period.

Building on this framework, Mercer introduced the concept of becoming a mother, highlighting maternal identity as a dynamic and evolving process rather than as a fixed outcome. From this perspective, maternal identity develops through emotional and behavioral engagement with pregnancy, the infant, and the changing family and social context (Mercer, 2004). Within this model, maternal role integration can be understood as the extent to which women are able to incorporate motherhood into their existing system of values, life projects, and social roles, while maintaining a sense of balance between caregiving demands and other meaningful life domains such as partnership, professional activity, and self-care.

Importantly, maternal role integration is conceptually distinct from related constructs frequently examined in the perinatal literature. While maternal identity refers specifically to the internalization and subjective recognition of oneself as a mother (Mercer, 2004; Rubin, 1984), and role balance reflects the perceived compatibility between motherhood and other life domains such as work, partnership, and self-care (Marks & MacDermid, 1996), maternal role integration represents a broader process that encompasses both elements within a coherent identity structure. In contrast, role strain refers to the experience of conflict or overload arising from competing role demands (Goode, 1960; Thoits, 1991). Thus, maternal role integration captures not only the presence of identity and balance, but also the extent to which these elements are successfully organized into a stable and meaningful sense of self during pregnancy.

Contemporary motherhood contexts pose specific challenges to this process of role integration. Increased female participation in the workforce, changing gender norms, and the diversification of family structures require many women to negotiate motherhood alongside multiple, and sometimes competing, roles (Han et al., 2024). Difficulties in integrating the maternal role within this broader identity system may give rise to experiences of ambivalence, self-doubt, and internal conflict, even in the absence of clinical psychopathology (Raneberg, 2025).

Although perinatal mental health has received increasing attention as a public health issue, most research has focused on the detection of emotional symptoms, such as depression and anxiety. Indeed, symptoms of psychological distress during pregnancy have been associated with a range of adverse outcomes for mothers and children (Howard et al., 2014; Stein et al., 2014). However, emotional symptoms represent only one aspect of women’s subjective experience during pregnancy and do not fully capture how women construct and integrate their maternal identity.

Several instruments have been developed to assess emotional processes related to the transition to motherhood, including measures of prenatal distress (Yali & Lobel, 1999) and maternal ambivalence (Martín-Sánchez et al., 2022), as well as widely used screening items for anxiety and depression (Cox et al., 1987; Zigmond & Snaith, 1983). While these instruments are valuable for identifying psychological symptoms and emotional difficulties, they do not directly assess the structural integration of the maternal role or the perceived balance between motherhood and other life roles.

Despite the centrality of maternal identity in theoretical models of the transition to motherhood, there is a notable lack of brief, conceptually specific instruments designed to assess maternal role integration during pregnancy, particularly in Spanish-speaking populations. Most existing measures either focus on emotional distress or are grounded in postpartum experiences, leaving the gestational period comparatively underexplored. To our knowledge, no brief instrument currently assesses maternal role integration as a distinct construct during pregnancy, highlighting a significant gap in the assessment of these processes (Cox et al., 1987; Yali & Lobel, 1999; Martín-Sánchez et al., 2022).

Difficulties in maternal role integration may also be expected to relate systematically to a broader network of psychological variables relevant to perinatal adjustment. Theoretically, lower integration of the maternal role may be associated with greater emotional distress, including depression, anxiety, and pregnancy-specific distress, as well as with more negative affect and lower positive affect, given the well-established links between role strain, identity conflict, and psychological distress (Goode, 1960; Thoits, 1991; Dunkel Schetter et al., 2022; Howard et al., 2014). At the same time, women who are able to incorporate motherhood into their sense of self while maintaining balance with other life domains may be more likely to report higher self-esteem, greater perceived social support, and higher life satisfaction, as suggested by identity theory and research on multiple-role integration and well-being (Burke & Stets, 2009; Thoits, 2012; Gardner et al., 2020). Maternal role integration may also be relevant to more specifically identity-related experiences during pregnancy, such as guilt and emotional ambivalence toward motherhood, which have been associated with difficulties in reconciling competing role expectations and internalized norms (Martín-Sánchez et al., 2022; Raneberg & MacCallum, 2025). Examining these associations is important not only to establish convergent and discriminant validity, but also to clarify the potential clinical relevance of the construct.

Therefore, the present study aimed to develop and validate the Maternal Role Integration Questionnaire—Pregnancy (MRIQ-P), an instrument designed to assess maternal role integration during pregnancy. The MRIQ-P conceptualizes this construct as comprising two complementary components: (a) maternal identity, defined as the extent to which women feel and recognize themselves as mothers, and (b) maternal role balance, defined as the perceived compatibility between motherhood and other significant life domains. Using a four-phase sequential design combining qualitative and quantitative methods, this study sought to produce a brief, reliable, and valid measure capable of capturing both a general factor of maternal role integration and specific dimensions of identity and role balance. In addition, the study examined associations between MRIQ-P scores and depression, anxiety, prenatal distress, affect, self-esteem, perceived social support, guilt, ambivalence, and life satisfaction, as well as its convergent, incremental, and group invariance validity.

## 2. Materials and Methods

This study adhered strictly to the ethical standards set forth in the Declaration of Helsinki and received formal approval from the ethics committee Rey Juan Carlos University. Participation was completely voluntary, and written informed consent was obtained from all participants before data collection began.

### 2.1. Participants and Procedures

To enhance methodological transparency, several additional procedures were implemented during the qualitative phase. Data collection followed a structured protocol: participants were recruited through primary healthcare centers and invited to take part in focus groups conducted in a private, quiet room to ensure confidentiality and psychological safety. A semi-structured guide with open-ended prompts was used to stimulate reflection on maternal identity and role balance, and all sessions were audio-recorded and transcribed verbatim.

Thematic analysis was conducted following Braun and Clarke’s framework, adopting an inductive approach. Two members of the research team independently coded the transcripts using NVivo 12, generating initial codes which were later refined through iterative comparison and grouping into higher-order themes. Regular consensus meetings were held to resolve discrepancies and ensure consistency in code application.

#### 2.1.1. Phase 1: Item Generation and Comprehension Review

##### Focus Groups (n = 17)

A total of 17 pregnant participants took part in the first phase (M = 31.82 years, SD = 4.73), recruited from two primary healthcare centers in Madrid, Spain. The eligibility criteria were as follows: (1) Spanish nationality, (2) pregnancy at any stage of gestation, (3) low obstetric risk, and (4) absence of diagnosed psychiatric disorders. No participants withdrew from the study.

The sessions were held between November and December 2024 and were facilitated by a psychologist experienced in qualitative research, with support from an observer responsible for field notes. Two focus groups were organized: one with 8 and another with 9 pregnant participants. Following Vogt et al. (2004), each session began with introductions, an explanation of the objectives and procedures (including audio recording and confidentiality), and participant self-presentations. The discussion was structured around open-ended prompts exploring how participants perceived and integrated the maternal role during pregnancy, encouraging detailed reflections and diverse viewpoints while maintaining a respectful and organized dialogue. Each meeting lasted approximately two hours and was audio-recorded with prior consent from the participants. The recordings were transcribed verbatim to ensure speaker identification. The research team conducted a systematic thematic analysis: transcripts were read repeatedly for familiarization, relevant segments were highlighted and coded, and the codes were progressively grouped into broader categories to capture recurring ideas and patterns. These categories informed the development of an initial draft of the MRIQ.

##### Cognitive Debriefing (n = 8)

This phase involved a convenience sample of eight pregnant participants recruited in February 2025 (M = 30.26 years, SD = 3.85), applying the same eligibility criteria as those used in the focus groups. Each participant independently reviewed the revised version of the MRIQ, which was developed based on the focus group findings and prior literature. They evaluated each item for clarity and relevance to the construct of maternal role integration, suggesting alternative wording when required. Feedback was systematically incorporated to refine ambiguous statements, ensuring that the questionnaire was comprehensible and contextually appropriate for the next validation stage (see Data Analysis for details).

#### 2.1.2. Phase 2: Expert Review of Content (n = 5)

Five professionals in psychology specializing in psychometrics, perinatal mental health, and maternal care participated in this stage (age range = 20–45 years). Experts were recruited through professional networks and asked to independently evaluate the revised version of the MRIQ obtained after cognitive debriefing. Each item was rated for relevance using a 4-point scale, and reviewers could provide written suggestions to improve clarity, propose additional content, or recommend removing items considered ambiguous or redundant. All evaluations were compiled and analyzed to calculate content validity indices and guide further refinement of the questionnaire.

#### 2.1.3. Phase 3: Examination of the Underlying Factor Structure (n = 256)

The scale was validated using a sample of pregnant women (N = 256). The participants had a mean age of 34.52 years (SD = 5.00; range = 20–45). Most were Spanish nationals (89.07%), with smaller proportions from Latin American and other European countries, primarily Argentina and Peru (1.18%), and several others (under 1% each). Over half were nulliparous (59.8%), and 83.2% reported no obstetric complications. Most of the participants showed high educational attainment (67.7% held higher education) and stable employment (66% were full-time). The average sleep duration was 7.47 h (SD = 1.28), with 86.3% of the participants reporting regular, good, or very good sleep quality. Most pregnancies were planned (74.2%), with a mean conception time of 8.68 months (SD = 14.48), and the average gestational age was 22.12 weeks (SD = 9.81). The sample included both primiparous and multiparous women. Specifically, 59.8% of participants were experiencing their first pregnancy, whereas 40.2% had experienced at least one previous pregnancy. Given the relevance of parity to maternal identity and role balance, measurement invariance across primiparous and multiparous women was examined.

All participants were recruited through online and community-based dissemination strategies, including social media platforms, hospitals, primary health care centers, and associations related to maternal and perinatal health. The inclusion criteria were Spanish nationality and pregnancy at any stage of gestation. Information about the study was distributed via digital channels and through collaborating institutions and associations, inviting eligible women to participate voluntarily. After confirming eligibility, participants accessed a secure online platform to complete the survey. The average completion time was approximately 15 min. All items were mandatory, preventing missing data. Responses were stored directly in a protected database for subsequent psychometric analyses.

The sample was randomly divided into two sub-samples. The first subsample (n = 83) completed exploratory factor analysis (EFA), which aimed to identify the underlying factor structure and refine the items. The sociodemographic characteristics of this subsample are summarized in Table 1. The second subsample (n = 173) completed confirmatory factor analysis (CFA), which was used to evaluate model fit, reliability, convergent and incremental validity, and group differences. Sociodemographic information of this subsample is presented in Table 1. Unequal subsample sizes were chosen following psychometric guidelines: smaller samples are acceptable for EFA, whereas CFA requires larger samples for stable estimation. Accordingly, approximately 30% of the total sample was assigned to the EFA and 70% (n = 173) to the CFA subsample.

The sample sizes used in the psychometric validation were grounded in methodological recommendations for factor analytic procedures. For the EFA, a subsample of 83 participants was deemed adequate given the characteristics of the model. Research indicates that samples between 50 and 100 can yield stable factor solutions when communalities are moderate to high, factor loadings exceed 0.60, and the number of factors is small (MacCallum et al., 1999; Lloret-Segura et al., 2014). These conditions were met in our analyses, where the refined 8-item model showed strong loadings and excellent fit indices.

For the CFA, a larger subsample of 173 participants was employed, aligning with recommendations suggesting that samples of 150–200 are appropriate for models with a limited number of latent factors and high loadings (Kline, 2016). The decision to allocate approximately 30% of the total sample to EFA and 70% to CFA follows cross-validation guidelines, which emphasize the need for a larger and independent subsample to test model stability. To evaluate potential biases or inaccuracies in the randomization process, we performed several statistical analyses, including independent samples t-tests and chi-square tests, to compare the sociodemographic variables between the two subsamples. These analyses assessed factors such as age, education level, income, employment status, sleep quality, and pregnancy-related characteristics of the participants. The findings revealed no statistically significant differences across any of the variables, indicating that the randomization was effective and that the subsamples were comparable. Furthermore, statistical comparisons confirmed the equivalence of the two subsamples, supporting the robustness of the split-sample validation approach.

### 2.2. Measures

The MRIQ-P was the sole instrument used in phases 1 and 2, and all subsequent measures were implemented exclusively in phase 3, together with the MRIQ-P.

#### 2.2.1. Maternal Role Integration

Maternal Role Integration was assessed with the Maternal Role Integration Questionnaire—Pregnancy version (MRIQ-P), a measure created specifically for the current research. The preliminary version comprised 10 items developed on the basis of qualitative evidence and expert review. Participants rated the frequency of specific cognitive experiences related to pregnancy during the past few weeks on a six-point Likert scale (1 = never; 6 = always). Details regarding the final set of items and the validated factor structure are reported in Section 3.

#### 2.2.2. Antenatal Depression

To measure depressive symptoms during pregnancy, we employed the Edinburgh Postnatal Depression Scale (EPDS; Cox et al., 1987) using the Spanish version developed by Garcia-Esteve et al. (2003), which has shown valid psychometric performance in samples of pregnant women in Spain (M. B. Vázquez & Míguez, 2019). The EPDS consists of 10 items assessing mood-related symptoms over the past week. Responses are provided on a 4-point Likert scale from 0 to 3, producing a total possible score of 0–30, where higher values represent more intense depressive manifestations. The initial Spanish validation reported acceptable internal reliability (α = 0.79). In our sample, internal consistency was excellent (Cronbach’s α = 0.89; McDonald’s ω = 0.90).

#### 2.2.3. Prenatal Distress

The Prenatal Distress Questionnaire (PDQ) (Yali & Lobel, 1999) is a self-assessment tool designed to assess stress specifically linked to pregnancy. It addresses maternal concerns about physical and emotional health, changes in relationships, the baby’s health, and the childbirth process. This measure comprises 12 items, each evaluated on a 5-point Likert scale (0 = not at all to 4 = very much), such that elevated scores indicate higher prenatal distress. We employed the Spanish version adapted and validated by Caparros-Gonzalez et al. (2019), who reported adequate internal consistency in Spanish pregnant samples (α = 0.74). In our sample, the PDQ similarly demonstrated solid reliability (Cronbach’s α = 0.81; McDonald’s ω = 0.82).

#### 2.2.4. Positive and Negative Affect

General emotional states were measured using the 20-item Positive and Negative Affect Schedule (PANAS; Watson et al., 1988), for which we employed the Spanish validated version developed by Sandín et al. (1999). The instrument evaluates two independent affective domains—positive affect (PA) and negative affect (NA)—each represented by 10 items. Respondents rate how strongly they have experienced each emotion on a 5-point Likert scale (1 = very slightly or not at all to 5 = extremely). Higher scores indicate greater levels of the respective emotional experience. Previous research has demonstrated satisfactory psychometric properties for both the original English version (Watson et al., 1988) and the Spanish adaptation (Sandín et al., 1999), supporting its factorial validity and internal consistency across cultural contexts. In the present study, internal consistency was good for positive affect (α = 0.86; ω = 0.86) and excellent for negative affect (α = 0.92; ω = 0.92).

#### 2.2.5. Self-Esteem

Global self-esteem was evaluated with the Rosenberg Self-Esteem Scale (RSES; Rosenberg, 1965), using the Spanish version validated by Martín-Albo et al. (2007). The scale is composed of ten items forming a single factor, with an equal number of positively and negatively worded statements. Participants rated each item on a 4-point Likert scale (1 = strongly disagree to 4 = strongly agree). Higher scores represent higher levels of self-esteem. Prior research with the Spanish adaptation has reported solid internal reliability in adult nonclinical populations (α = 0.85). In our sample of pregnant women, reliability indices were similarly strong (α = 0.89; ω = 0.89).

#### 2.2.6. Perceived Social Support

The Multidimensional Scale of Perceived Social Support (MSPSS) was employed to assess perceived social support, using the Spanish adaptation by Landeta and Calvete (2002). This instrument measures the level of support individuals perceive from three sources: family, friends, and significant others. The original scale comprises 12 items, each rated on a 7-point Likert scale from 1 (very strongly disagree) to 7 (very strongly agree), allowing for the calculation of both subscale and total scores. To reduce participant burden while preserving conceptual coverage, we selected two items from each subscale. Item selection was guided by their psychometric strength (i.e., highest factor loadings reported in previous validation studies) and their conceptual representativeness of each support source. Thus, the short version retained the most robust indicators of family, friend, and significant-other support while maintaining balanced coverage of the three domains. In the present study, analyses were conducted using the total score of this abbreviated version. This customized shorter version demonstrated good internal consistency in our sample (α = 0.87, ω = 0.87).

#### 2.2.7. Dispositional Guilt

We assessed the predisposition to experience excessive or inappropriate guilt using the subset of five items constituting Factor 2 of the Guilt Feelings Scale (SC-35; Zabalegui, 1993), originally validated in Spain. This factor captures guilt reactions that occur in contexts where such feelings are unjustified or exaggerated relative to the situation. Participants rated each item on a 4-point Likert scale (1 = totally false to 4 = totally true). Higher scores indicate a greater tendency to feel guilt. Reliability for this abbreviated set was strong in the present study (α = 0.85; ω = 0.86).

#### 2.2.8. Emotional Ambivalence

The Maternal Ambivalence Scale (MAS) was employed to assess the mixed emotions surrounding motherhood, such as happiness and sadness, using the Spanish adaptation by Martín-Sánchez et al. (2022). This scale measures the conflicting reactions towards being a mother and their effects at the cognitive, affective, and behavioral levels. To measure this ambivalence, the scale seeks the presence of doubts, the degree of conviction, and the use of coping strategies. The Spanish version of the scale has 14 items rated on a 4-point Likert scale, ranging from 1 (completely disagree) to 4 (completely agree). The Spanish validation showed good internal consistency, with α = 0.83 for doubts and α = 0.70 for conviction. In our sample, the consistency was good for doubts (α = 0.86, ω = 0.89) and conviction (α = 0.82, ω = 0.83).

#### 2.2.9. General Depression and Anxiety

To assess depression and anxiety levels, we utilized the Spanish version of the Hospital Anxiety and Depression Scale (HADS) (Zigmond & Snaith, 1983), validated in a Spanish sample by Herrero et al. (2003). This instrument measures symptoms over the preceding week, avoiding symptoms that could be caused by physical causes such as insomnia. This 14-item self-report instrument has seven items for anxiety and seven items for depression on a 5-point Likert scale from 1 (for example, as much as I always do) to 5 (for example, not at all), yielding a maximum score of 21 for each subscale. The Spanish adaptation has shown good psychometric properties, with α = 0.9 for the full scale, α = 0.84 for the depression scale, and α = 0.85 for the anxiety scale. In our sample, the internal consistency was acceptable for anxiety (α = 0.72 and ω = 0.75) and depression (α = 0.71 and ω = 0.70).

#### 2.2.10. Life Satisfaction

To assess global life satisfaction, we employed the Spanish version of the Satisfaction with Life Scale (SWLS) (Diener et al., 1985), as validated by C. Vázquez et al. (2013). This unidimensional instrument consists of five items designed to measure life satisfaction as a global cognitive process. Each item was rated on a 7-point Likert scale ranging from 1 (strongly disagree) to 7 (strongly agree). The Spanish version of the study had good internal consistency (α = 0.8). In our sample, the scale demonstrated strong internal consistency (α = 0.88 and ω = 0.88).

### 2.3. Data Analysis

During the item generation stage, following Vogt et al.’s (2004) recommendations, focus groups were convened to collect comprehensive data. The data were examined using NVivo 12 through thematic and inductive methods to identify recurring themes related to maternal role integration during pregnancy. The codes were refined and organized into broader themes that captured the psychological and contextual dimensions involved in the construction of maternal identity and the perceived balance between motherhood and other life roles. These themes, derived from the data, served as the primary foundation for creating items aimed at capturing maternal identity and role balance during pregnancy. By integrating insights from both sources and adhering to established scale development guidelines (Hinkin, 1998), the research team crafted items that were clear and straightforward, accurately reflecting the participants’ language and experiences. The items were designed to be unambiguous, non-leading, and easy to comprehend, avoiding unnecessary reverse-scoring formats. This meticulous approach resulted in the initial draft of the MRIQ-P, which was intended to comprehensively capture the concept. Subsequently, during cognitive debriefing, pregnant women evaluated each item for clarity and relevance on a 7-point Likert scale and suggested wording changes. Items that scored below 5 in terms of clarity or relevance were earmarked for modification or elimination. This process ensured that the MRIQ-P version post-cognitive debriefing was easy to understand and possessed strong face validity.

Phase 2, which focused on expert-based content evaluation, involved an independent review by a panel of five specialists in psychometrics, perinatal psychology, and maternal health. These experts assessed the post-cognitive debriefing version of the MRIQ-P and evaluated each item for relevance using a 4-point scale. Consistent with previous studies (Polit et al., 2007), the Content Validity Index (CVI), a standard metric for assessing content validity, was calculated. The item-level CVI (I-CVI) was determined by dividing the number of experts who rated an item as 3 (quite relevant) or 4 (highly relevant) by the total number of experts who rated it. The CVI scores ranged from 0 to 1, with a score of 1 indicating unanimous expert agreement. Items with an I-CVI below 1.00 were identified for potential revision or removal, following the recommendations of Polit et al. (2007). Additionally, a scale-level CVI (S-CVI) was calculated by averaging the I-CVI scores for all items. Items with low CVI scores were rephrased or eliminated. Experts were also encouraged to provide concise qualitative feedback to enhance the item wording and address any potential clarity issues alongside the quantitative assessment. The quantitative evaluation using I-CVI and S-CVI, combined with expert feedback, resulted in an expert-refined version of the MRIQ-P.

The analytical strategy followed established guidelines for scale development. An Exploratory Factor Analysis (EFA) was first conducted because the MRIQ-P represents a newly developed instrument with no previously validated structure. As recommended in psychometric literature (Fabrigar et al., 1999; Lloret-Segura et al., 2014), EFA is the most appropriate method when the underlying dimensionality is unknown or only theoretically inferred. This approach allows the identification of latent factors and the evaluation of item behavior without imposing strong a priori constraints. An exploratory factor analysis (EFA) was conducted on the expert-refined MRIQ-P to explore its dimensional structure. Univariate normality was checked through skewness and kurtosis thresholds (±2 and ±3, respectively), complemented by histogram and Q–Q plot inspections. Multivariate normality was evaluated using Mardia’s test (*p* ≤ 0.05). The suitability of the data for factor analysis was verified using the Kaiser–Meyer–Olkin (KMO) statistic (>0.80) and Bartlett’s test of sphericity (*p* < 0.05). Following the guidelines of Lloret-Segura et al. (2014), several procedures were combined to determine the appropriate number of factors: Horn’s parallel analysis (comparing real versus simulated eigenvalues), the scree plot (identifying discontinuities), and Velicer’s MAP test (selecting the solution with the lowest average squared partial correlations). Because the sample size was relatively small (n = 83) and the data approximated normality, the EFA was performed using Pearson correlations together with MINRES extraction and Promax rotation, a combination recommended for small-sample conditions to avoid limitations of maximum likelihood estimation (Fabrigar et al., 1999; Lloret-Segura et al., 2014). Items were flagged for potential removal if they exhibited communalities below 0.40, cross-loading disparities smaller than 0.15, or cross-loading values above 0.32 across multiple factors (Hair et al., 2019; Worthington & Whittaker, 2006). Model adequacy was assessed through RMSR, RMSEA, and CFI indices (Hair et al., 2019; Lloret-Segura et al., 2014).

A Confirmatory Factor Analysis (CFA) was subsequently performed on an independent subsample to test the stability of the factor structure identified in the EFA. Using CFA after EFA is considered best practice in modern scale validation because it enables the comparison of theoretically plausible models, the assessment of global and local fit, and the examination of factorial complexity under more rigorous assumptions (Brown, 2015). Testing a bifactor model alongside one- and two-factor models allowed us to determine whether maternal role integration was best represented by a general factor, by distinct subdimensions, or by a hierarchical configuration. The revised MRIQ-P was reassessed using a sample of 173 participants. Mardia’s test indicated that the data did not conform to a normal distribution, necessitating item ranking. Consequently, the WLSMV method was used. Notably, the χ^2^ statistic of this method may erroneously reject well-fitting models when dealing with ranked items and small sample sizes. Therefore, additional fit indices, such as TLI, CFI, NFI, SRMR, and RMSEA, were used to assess model fit. Typically, a model is considered a good fit if the CFI/TLI is 0.90 or higher, the SRMR is 0.10 or lower, and the RMSEA is 0.08 or lower. However, Hu and Bentler (1999) propose more rigorous criteria: CFI/TLI/NFI should be 0.95 or higher, SRMR should be 0.08 or lower, and RMSEA should be 0.06 or lower. The model’s local fit was evaluated using factor loadings (λ) and item reliability (R^2^), with satisfactory item performance indicated by λ values ≥ 0.70 and R^2^ values ≥ 0.50. Cronbach’s alpha (α) and McDonald’s omega (ω) were calculated to assess internal consistency, with omega providing greater accuracy when item loadings varied. A value of ≥0.70 for both metrics indicated good reliability. Finally, various model structures were tested, and the EFA-derived solution was compared with unidimensional, bifactor, and second-order models to identify the optimal fit for the data.

This sequential use of EFA and CFA is widely recommended in scale development to ensure both the empirical identification of latent structures and the confirmation of their stability across independent samples (Fabrigar et al., 1999; Brown, 2015; Kline, 2016).

Independent samples t-tests were performed to explore potential differences in both the total MRIQ-P score and its dimensions, considering a relevant contextual variable: the presence or absence of a prior anxiety diagnosis. As the assumption of normality was not met (assessed using the Shapiro–Wilk test), a non-parametric approach was adopted. Specifically, the Mann–Whitney U test was used to compare the two groups. To quantify the magnitude of the difference, the effect size was calculated using Glass’ Rank-biserial correlation coefficient (r_b_). Effect size interpretation followed the most common guidelines: <0.05 = very small, 0.10 = small, 0.20 = moderate, and ≥0.30 = large (Funder & Ozer, 2019).

Pearson’s bivariate correlations were used to evaluate the convergent and discriminant validity of the MRIQ-P scores in relation to various theoretically associated psychological constructs. These constructs encompassed personality variables such as self-esteem, positive and negative affect, and maternal ambivalence, with particular emphasis on the dimensions of doubt and conviction. Additionally, well-being variables, including general anxiety, depression, and prenatal distress, were considered. Significant and sufficiently large correlations indicated convergent validity (Campbell & Fiske, 1959), whereas correlations below 0.90 demonstrated discriminant validity (Kline, 2011).

Incremental validity was examined through a series of multiple linear regression models aimed at identifying whether the MRIQ-P dimensions provided additional explanatory power for antenatal depression, dispositional guilt, and life satisfaction after controlling for sociodemographic factors, contextual influences (such as maternal age, trimester, and gestational complications), and other psychological variables (including perceived social support and cognitive fusion). Binary covariates were dummy-coded, and all predictors were introduced simultaneously using the enter procedure, enabling an estimation of the unique variance attributable to the MRIQ-P scores.

We performed measurement invariance analyses to determine whether the scores were consistent between women in their first pregnancy and those who had been pregnant before. We utilized four levels of invariance as outlined by (Dimitrov, 2010): (M1) configural (identical latent structure), (M2) metric (incorporating equal factor loadings), (M3) scalar (incorporating equal item intercepts), and (M4) strict (incorporating equal item residuals). Achieving metric invariance is crucial for making valid group comparisons. We assessed fit indices by analyzing changes in CFI, TLI, and RMSEA across models (from M1 to M5), with criteria for non-invariance defined as ΔCFI and ΔTLI ≤ 0.010, and ΔRMSEA ≥ 0.015 (Chen, 2007). Considering the ongoing debate in the literature about these guidelines, we also examined the differences in χ2 and SRMR as additional measures (Putnick & Bornstein, 2016).

Quantitative analyses were conducted using R (version 4.4.3) with the following packages: *psych*, *MVN*, *REdaS*, *dplyr*, *effsize*, *REdaS*, *userfriendlyscience*, *EFAtools*, *e1071*, *tidyverse*, *grid*, *tibble*, *ggplot2*, *lavaan*, *haven*, *lavaangui*, *BifactorIndicesCalculator*, *MBESS*. Qualitative analyses were executed using NVivo 12 (QSR International) to perform thematic coding of focus group data, which was further supplemented by a manual review of participant ratings and comments during item evaluation procedures, including cognitive debriefing and expert panel assessment.

## 3. Results

### 3.1. Item Generation and Comprehension Review

Qualitative analysis of the two focus groups conducted with pregnant women revealed key experiential domains related to the process of maternal role integration during pregnancy. Rather than focusing on emotional symptoms, participants’ narratives emphasized identity-related and role-related processes involved in becoming a mother. Five interrelated thematic domains emerged from the data: (1) emotional internalization of the maternal role, including the subjective sense of “feeling like a mother” and recognizing pregnancy as part of one’s self-concept; (2) continuity and change in personal identity, reflecting negotiations between the emerging maternal role and pre-existing identities (e.g., partner, professional, daughter, or individual self); (3) perceived balance between motherhood and other life domains, such as work, couple relationships, and personal projects; (4) expectations and perceived social norms surrounding motherhood, including internalized ideals and perceived external pressures; and (5) anticipatory reflections about future caregiving demands and role responsibilities.

These thematic domains were established through consensus-based coding and served as the conceptual foundation for item development. Based on these findings, the research team generated an initial pool of 19 items designed to capture the emotional and cognitive aspects of maternal role integration. Item wording was derived directly from the participants’ language to preserve ecological validity and conceptual clarity. This process was further informed by a focused review of the theoretical and empirical literature on maternal identity development, role integration, and role balance during the transition to motherhood. The items were formulated to be emotionally resonant while avoiding symptom-based or clinical phrasing. Responses were recorded using a 6-point Likert scale ranging from 1 (never) to 6 (always).

During the cognitive debriefing phase, the participants evaluated the clarity and relevance of each item using a 7-point rating scale. Overall, most items received high ratings, with mean scores above 5.0 for clarity and relevance. Qualitative feedback, however, indicated some redundancy between items addressing closely related aspects of identity internalization and role continuity. In addition, several items referring to comparisons with previous pregnancies were perceived as less relevant by nulliparous women and were therefore considered unsuitable for the target populations. Based on these quantitative ratings and qualitative comments, the research team refined the item pool by removing redundant items and excluding those with limited applicability to the current study. As a result, the post–cognitive debriefing version of the Maternal Role Integration Questionnaire (MRIQ) retained 13 items across four conceptual domains, which were subsequently submitted for expert-based content evaluation.

### 3.2. Expert-Based Content Evaluation

Upon evaluation, the MRIQ-P items were determined to possess generally high content validity indices (CVIs). Five experts assessed the 13 items for relevance using a 4-point scale, resulting in item-level CVI (I-CVI) values ranging from 0.33 to 1.00. The majority of items achieved unanimous agreement among the experts (I-CVI = 1.00); however, three items received significantly lower ratings, with only one expert considering them quite or highly relevant (I-CVI = 0.33), which was below the threshold for potential revision or removal (Polit et al., 2007). Consequently, these three items were excluded from the scale owing to their CVI scores and qualitative feedback from the experts, which highlighted issues such as insufficient focus on pregnancy, conceptual overlap across dimensions, and a future-oriented perspective that diminished their immediate relevance to the construct. As a result, the expert-refined version of the MRIQ-P retained 10 items for subsequent exploratory factor analysis.

### 3.3. Examination of the Underlying Factor Structure (EFA)

**Figure 1:**
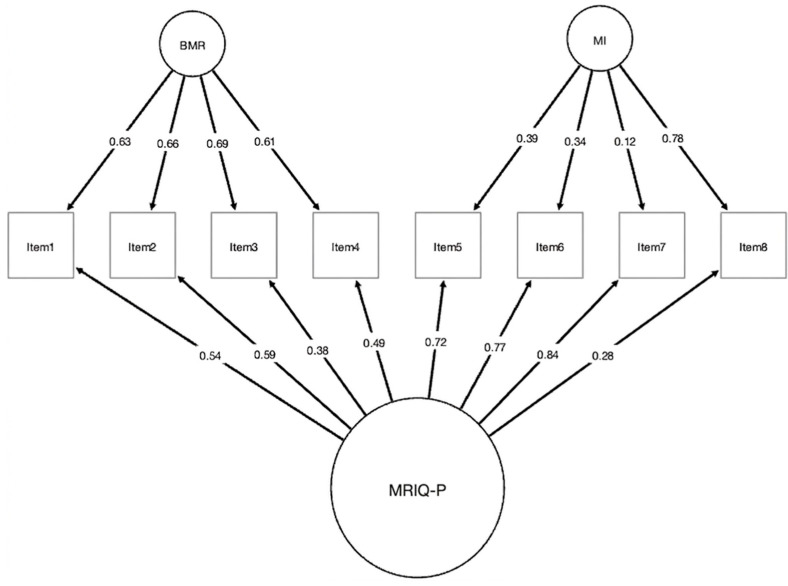
Final bifactor confirmatory factor analysis (CFA) model of the MRIQ-P. Note. Standardized factor loadings are shown. MRIQ-P = Maternal Identity Role Questionnaire—Pregnancy; MI = Maternal Identity; BMR = Balance of the Maternal Role.

The expert-refined MRIQ-P, consisting of 10 items, satisfied the prerequisites for factor analysis, evidenced by a meritorious KMO value of 0.871 and a significant Bartlett’s test (χ^2^ = 1153.97; df = 45; *p* < 0.001). Both the scree plot and MAP test supported a two-factor solution, aligning with the thematic analysis, whereas the parallel analysis suggested three factors. Consequently, a two-factor model was assessed. The EFA results are presented in the left section of Table 2 and illustrated in Figure 1. The two identified factors accounted for 53.3% of the variance, demonstrated a satisfactory model fit (SRMR = 0.030; RMSEA = 0.065, TLI = 0.995), and the mean item complexity was 1.1. However, two items exhibited communalities below 0.40 (see items 6 and 9, Table 2). Consequently, these two items were removed from the scale. A revised EFA was conducted on an 8-item structure (four per factor). The KMO value remained adequate at 0.80, and Bartlett’s test was again significant (χ^2^ = 287.099; df = 28; *p* < 0.001). As before, the scree plot, MAP test, and parallel analysis suggested two factors. The right section of Table 2 displays the factor loadings, communalities, and complexity for this refined solution, which exhibited a cleaner structure with loadings from 0.49 to 0.88, no significant cross-loadings, and an average item complexity of 1.1. The two factors collectively accounted for 56.5% of the total variance, and the model exhibited an excellent fit (SRMR = 0.03; RMSEA = 0.01, TLI = 0.98). Overall, these results support the adoption of this refined 8-item configuration as the most parsimonious and theoretically coherent solution (see Figure 1).

These findings indicate that the retained items showed strong and consistent associations with their respective factors, supporting the structural coherence of the scale and the adequacy of the two-factor solution. The absence of substantial cross-loadings further reinforces the conceptual distinctiveness of the identified dimensions.

The CFA compared three theoretically relevant configurations estimated using the WLSMV method: a one-factor model (assuming a single latent dimension), a correlated two-factor model (replicating the EFA outcome with MI and BMR as distinct but related dimensions), and a bifactor model (comprising one general factor and two specific dimensions, MI and BMR). The fit indices in Table 3 indicate that the one-factor model had an inadequate fit, confirming that maternal role identity cannot be explained by a single latent dimension. The two-factor model showed an acceptable fit, supporting the multidimensional nature of the construct. However, the bifactor model provided the best overall fit according to the CFI, TLI, RMSEA, and SRMR criteria, and chi-square difference tests favored the bifactor solution over the two-factor alternative. Conceptually, the bifactor structure offers the advantage of modeling both the overarching maternal role identity and the unique contributions of MI and BMR, which aligns with the MRIQ’s theoretical framework. Therefore, the bifactor model was retained as the final measurement model in this study.

In the finalized bifactor structure, the two specific factors identified through the exploratory and confirmatory procedures were labeled according to the thematic content of their items. The Maternal Identity (MI) factor reflects emotional engagement and the degree to which the maternal role is internalized, whereas the Balance of the Maternal Role (BMR) factor captures the perceived capacity to harmonize motherhood with other life responsibilities. These labels—and their corresponding acronyms—were used consistently in all subsequent analyses and tables for clarity. The general factor demonstrated an explained common variance (ECV) of 0.53, indicating that over half of the shared variance among items is attributable to a common underlying construct. This supports the presence of a dominant general factor driving responses across the scale. Nevertheless, the specific factors also accounted for important portions of common variance, with ECV values of 0.32 for MI and 0.62 for BMR, showing that BMR in particular represents a distinct and meaningful facet of the maternal role experience.

Reliability analyses further supported the robustness of the bifactor model. Total omega (ω) values were high for the general factor (ω = 0.93) and for both specific dimensions (MI = 0.89; BMR = 0.89). Hierarchical omega (ωH) indicated that 0.64 of reliable variance was attributable specifically to the general factor, while MI and BMR accounted for 0.25 and 0.56, respectively. These results confirm that although the general factor is influential, the specific factors—especially BMR—provide additional, non-redundant information. Cronbach’s alpha values based on polychoric correlations demonstrated excellent internal consistency across the instrument. The total scale showed an alpha of 0.96, while both MI and BMR exhibited exceptionally high reliability (α = 0.98 for each), underscoring the precision and coherence of the measure and its subdimensions.

As shown in Table 4, participants with an anxiety diagnosis scored significantly lower on all MRIQ-P dimensions compared to those without such a diagnosis (all *p* < 0.05), with small-to-moderate effect sizes (rank-biserial correlations ranging from −0.168 to −0.215). Specifically, differences were observed in Maternal Identity (MI), Balance of the Maternal Role (BMR), and total scores, suggesting that anxiety symptoms are associated with a less integrated maternal role identity and perceived balance. These findings indicate a consistent pattern of reduced scores among women with anxiety, highlighting the potential impact of anxiety on maternal role identity.

Regarding convergent and discriminant validity, both the overall MRIQ-P score and its dimensions (Maternal Identity and Balance of the Maternal Role) showed significant associations with external psychological variables (see Table 5). Specifically, higher MRIQ-P scores were positively correlated with self-esteem and positive affect and negatively correlated with negative affect, maternal ambivalence (doubts and conviction), general anxiety, general depression, and prenatal distress. The correlations among the MRIQ-P dimensions and the total score were high but not redundant, supporting their conceptual distinction. This pattern of relationships provides empirical evidence for the MRIQ-P’s convergent and discriminant validity.

Hierarchical regression analyses were conducted to evaluate the incremental contribution of the MRIQ-P dimensions in predicting antenatal depression, dispositional guilt, and life satisfaction (see Table 6). After controlling for age, gestational age, and pregnancy complications (Step 1), as well as perceived social support and cognitive fusion (Step 2), the inclusion of MRIQ-P dimensions in Step 3 accounted for an additional 2.0% of the variance in antenatal depression (*p* < 0.05), 4.6% in dispositional guilt (*p* < 0.01), and 4.0% in life satisfaction (*p* < 0.01). In the final models, BMR was identified as a significant negative predictor of antenatal depression (*β* = –0.179, *p* < 0.05) and dispositional guilt (*β* = –0.290, *p* < 0.001), whereas MI uniquely predicted life satisfaction (*β* = 0.154, *p* < 0.05). The comprehensive models explained 54.7% of the variance in antenatal depression, 34.7% in dispositional guilt, and 37.8% in life satisfaction, highlighting the significance of maternal role identity dimensions in psychological outcomes during pregnancy.

The invariance analyses are presented in Table 7. Metric invariance was supported across all comparisons between women experiencing their first pregnancy and those who had previous pregnancies. Given that metric invariance was achieved, partial invariance analysis was not considered. Furthermore, scalar invariance was established, allowing for meaningful comparisons of latent means between groups. Finally, strict invariance was supported, indicating that the observed scores could be compared across groups without bias. These results confirm that the MRIQ scale operates equivalently, regardless of whether it is the first pregnancy.

## 4. Discussion

The present study aimed to develop and validate a novel questionnaire designed to assess maternal role integration during pregnancy, with a particular focus on maternal identity and role balance. Overall, the findings support the psychometric soundness of the Maternal Role Integration Questionnaire (MRIQ), demonstrating adequate internal consistency, a theoretically coherent factor structure, and meaningful associations with related psychological constructs. The results indicate that maternal role integration can be conceptualized as a multidimensional yet cohesive construct that operates consistently across women experiencing their first pregnancy and those with previous pregnancies.

These findings extend existing identity and role theories by suggesting that successful adaptation to motherhood during pregnancy involves not only the development of a maternal identity, but also the integration of this role within a broader and coherent identity system. This perspective is consistent with identity theory and multiple-role frameworks, which emphasize that psychological adjustment depends on the compatibility and integration of roles rather than their mere accumulation (Burke & Stets, 2009; Thoits, 2012).

Consistent with the proposed theoretical model, the results supported a hierarchical structure comprising a general factor of maternal role integration alongside two specific dimensions: Maternal Identity (MI) and Balance of the Maternal Role (BMR). This structure reflects the inherent complexity of the maternal role, which involves simultaneously “becoming a mother” while negotiating continuity and change with previously established identities (e.g., partner, professional, daughter, or friend). MI captures the emotional and symbolic internalization of the maternal role, including the subjective sense of “feeling like a mother” and recognizing pregnancy as part of one’s self-concept. In contrast, the BMR reflects the perceived ability to integrate motherhood with other valued life domains, maintaining a sense of balance and continuity rather than experiencing motherhood as an exclusive, overwhelming, or incompatible role.

This interpretation aligns with role identity and multiple-role frameworks, which emphasize that psychological well-being is facilitated when new roles are integrated into a coherent identity structure rather than experienced as conflicting with existing roles (Burke & Stets, 2009; Thoits, 1983, 2012). The superiority of the bifactor model suggests that maternal role integration operates as a broad organizing construct encompassing more specific experiences related to identity consolidation and role balance. From a practical standpoint, this structure allows researchers to use a global MRIQ score when a general index of maternal role integration is required, while preserving the interpretive value of the MI and BMR subscales for more fine-grained analyses.

Evidence for convergent and divergent validity further substantiated the robustness of the MRIQ. Both MI and BMR were positively associated with self-esteem and positive affect and negatively associated with negative affect, general anxiety, and general depression. These findings suggest that a more integrated maternal role is linked to greater psychological well-being (Adler et al., 2016). Importantly, the pattern of associations revealed a meaningful differentiation between the two dimensions. BMR showed stronger associations with indicators of emotional distress, including prenatal distress, anxiety, and depression, consistent with the theoretical models of role strain and role conflict. Difficulties in reconciling motherhood with other responsibilities may lead to experiences of overload, guilt, or perceived failure when women feel unable to meet competing internal or external expectations (Goode, 1960; Thoits, 1991).

By contrast, MI was more strongly related to variables such as maternal conviction and life satisfaction, suggesting that emotional identification with the maternal role may function as a source of meaning and psychological protection during the transition to motherhood. This differentiation aligns with prior research indicating that identity affirmation and role meaning are more closely associated with well-being, whereas role conflict and imbalance are more directly linked to distress (Simon, 1995). Together, these findings support the importance of conceptualizing maternal role integration as a multidimensional construct, in which different components are differentially associated with distinct aspects of emotional adjustment.

Additional support for construct validity was provided by the association with maternal ambivalence. Higher MRIQ scores (particularly on the MI dimension) were associated with lower levels of maternal doubts and higher levels of conviction, indicating that greater role integration is linked to reduced internal conflict and greater clarity regarding the maternal role. These findings suggest that the MRIQ captures the central processes involved in resolving ambivalence and integrating potentially contradictory feelings about pregnancy and motherhood. Divergent validity was also supported, as associations with sociodemographic variables such as age, educational level, gestational age, sleep duration, and parity were generally weak or nonsignificant, indicating that the instrument primarily assesses psychological processes rather than demographic or situational characteristics.

A further relevant finding concerned the differences between women with and without a prior diagnosis of anxiety. Pregnant women reporting a history of anxiety scored significantly lower on MI, BMR, and the overall MRIQ, with small-to-moderate effect sizes. This pattern is consistent with previous research indicating that anxiety during pregnancy is associated with heightened self-criticism, intolerance of uncertainty, and difficulties in positive self-referential processing, which may interfere with the consolidation of a positive maternal identity (Dunkel Schetter et al., 2022; Fairbrother et al., 2016; Field, 2017). Lower maternal role integration among anxious women may reflect greater difficulty envisioning themselves as competent mothers, increased perceived incompatibility between motherhood and other life domains, or a tendency to interpret normative doubts as signs of inadequacy. From a clinical perspective, these findings suggest that assessing maternal role integration may help identify women who, beyond experiencing anxiety symptoms, are at risk of a more fragile or conflicting identity reorganization during pregnancy.

Evidence for construct specificity was further supported by the incremental validity analyses. After controlling for established predictors of perinatal adjustment, such as perceived social support and cognitive fusion, the MRIQ dimensions explained additional variance in antenatal depression, dispositional guilt, and life satisfaction. Notably, BMR uniquely predicted antenatal depression and dispositional guilt, suggesting that difficulties in managing and integrating maternal responsibilities contribute to emotional distress beyond the effects of cognitive rigidity and available social resources. In contrast, MI emerged as the strongest predictor of life satisfaction, reinforcing the idea that a consolidated maternal identity is closely linked to positive global evaluations of one’s life trajectory (Gardner et al., 2020). The fact that not all MRIQ dimensions predicted all outcomes further underscores the value of a multidimensional approach to maternal-role integration.

From an applied perspective, the MRIQ may be particularly useful in research and preventive contexts, offering a means to identify early difficulties in maternal role integration during pregnancy. By focusing on identity and role balance rather than symptom severity, the instrument complements existing measures of perinatal mental health and contributes to a more nuanced understanding of women’s subjective experiences during the transition to motherhood. This focus may facilitate the development of interventions aimed at supporting identity integration and role balance, thereby promoting psychological well-being during pregnancy.

## 5. Limitations and Future Directions

The present study has several limitations that should be acknowledged. First, the cross-sectional design precludes any causal interpretation of the observed associations. It remains unclear whether difficulties in maternal role integration lead to poorer emotional well-being, whether diminished well-being interferes with the consolidation of maternal identity, or whether these processes mutually reinforce one another. Future research using longitudinal, diary, and experience-sampling designs is needed to clarify the temporal dynamics of maternal role integration across pregnancy and into the postpartum period.

Second, the exclusive reliance on self-report measures represents an additional limitation. Although subjective experience is central to the assessment of maternal identity and role integration, self-report methods are inherently susceptible to social desirability, recall bias, and shared method variance and may not fully capture the implicit, behavioral, or relational aspects of this construct. Future studies would benefit from adopting multimethod approaches, including partner or clinician reports, observational methods, or behavioral tasks related to role negotiation and decision-making. In this regard, it is also important to note that the full MSPSS was not administered; only the abbreviated 6-item version was used, which prevented us from examining its empirical correlation with the original 12-item scale.

Third, although item development followed a rigorous and theory-informed process involving qualitative work with pregnant women and a review by a multidisciplinary expert panel, limitations related to content and ecological validity should be considered. Certain facets of maternal identity and role integration may not have been fully captured, such as the emotional negotiation of leaving behind previous roles (e.g., as a daughter), experiences associated with unplanned or undesired pregnancies, medically assisted reproduction, or culturally specific expectations surrounding motherhood. Future refinement of the MRIQ-P may involve expanding item content through qualitative research in more diverse samples, including women from different cultural backgrounds, socioeconomic contexts, family structures, and gender identities.

Finally, the generalizability of the findings is limited by characteristics of the sample. Participants were primarily pregnant women residing in Spain, recruited through online dissemination strategies, and were characterized by relatively high levels of education and employment stability. Consequently, the findings may not be generalizable to other populations, such as women with fewer socioeconomic resources, migrants, adolescents, women living in rural settings, or those facing higher levels of contextual adversity. Moreover, maternal role integration is likely relevant across a broader range of reproductive and caregiving contexts, including women contemplating motherhood, those attempting conception, women in the postpartum period, and non-gestational mothers in same-sex couples. Future research should examine the applicability and measurement invariance of the MRIQ across these groups and in diverse cultural contexts to further establish its robustness and utility.

## 6. Clinical Implications

Beyond its psychometric robustness, the MRIQ-P has several relevant clinical implications for perinatal care. First, it offers a brief and conceptually focused tool to assess maternal role integration during pregnancy, an aspect of psychological functioning that is not directly captured by traditional symptom-based screening instruments such as measures of depression or anxiety. While existing tools are essential for detecting emotional distress, they do not assess how women are integrating motherhood into their identity structure or negotiating a balance between caregiving and other life domains.

The present findings suggest that lower maternal role integration (particularly difficulties in balancing the maternal role) is associated with greater antenatal depression and dispositional guilt, even after controlling for established psychological predictors such as perceived social support and cognitive fusion. This indicates that maternal role integration may represent a distinct vulnerability factor, with potential relevance for early identification of women at risk of emotional maladjustment during pregnancy.

From a preventive perspective, the MRIQ-P could be incorporated into routine psychological assessment in obstetric, midwifery, or primary care settings to identify women experiencing identity-related difficulties before the emergence of clinically significant symptoms. Women who report low levels of maternal identity consolidation or role balance may benefit from targeted interventions focused on identity clarification, expectation management, role negotiation within the couple or workplace, and normalization of ambivalence. Furthermore, the bifactor structure of the MRIQ-P allows clinicians to use either a global score of maternal role integration or to examine specific dimensions (Maternal Identity and Balance of the Maternal Role), depending on the clinical objective. For example, low scores in Maternal Identity may indicate difficulties in emotional internalization of the maternal role, whereas low scores in Balance of the Maternal Role may reflect perceived overload, incompatibility between roles, or anticipatory role strain. This differentiation may inform individualized intervention planning. Finally, given the demonstrated measurement invariance across primiparous and multiparous women, the MRIQ may be used to monitor maternal role integration across different reproductive trajectories. Longitudinal application of the instrument may also help evaluate the effectiveness of preventive or therapeutic programs aimed at supporting psychological adjustment during pregnancy.

Overall, the MRIQ complements existing perinatal screening tools by addressing identity and role-related processes that are central to the transition to motherhood, and that may precede, accompany, or even underlie emotional symptomatology. Its integration into perinatal healthcare settings may contribute to a more comprehensive and preventive model of maternal mental health assessment.

## 7. Conclusions

This study provides initial evidence for the Maternal Role Integration Questionnaire (MRIQ-P) as a brief and psychometrically robust instrument for assessing how women integrate their maternal role during pregnancy. The findings support a bifactor structure comprising a general factor of maternal role integration and two specific dimensions (Maternal Identity and Balance of the Maternal Role) with excellent internal consistency and good indices of convergent, discriminant, incremental, and measurement invariance. Importantly, the MRIQ captures identity- and role-related processes that are meaningfully associated with perinatal mental health but are not reducible to symptoms of anxiety or depression, thereby offering a complementary perspective to existing screening tools.

From an applied perspective, the MRIQ may be useful in both research and clinical contexts. In research settings, it provides a quantitative operationalization of maternal role integration that can be incorporated into longitudinal models examining risk and resilience during the transition to motherhood, as well as into intervention studies targeting perinatal adjustment. Clinically, the instrument may help identify women who, despite reporting relatively low levels of emotional symptoms, experience difficulties integrating the maternal role or maintaining a satisfactory balance between motherhood and other life domains. Such difficulties may represent a vulnerability factor for later emotional problems and could be addressed through preventive or therapeutic approaches focused on identity work, role negotiation, and promoting realistic and flexible maternal expectations.

More broadly, these findings highlight the importance of considering maternal role integration as a central psychological process in perinatal care. Interventions aimed at strengthening maternal identity through reflective dialogue about expectations and values (or group-based spaces that normalize ambivalence) and at supporting a more balanced integration of motherhood with work, couple, and personal projects may contribute not only to reducing distress but also to enhancing life satisfaction and the overall meaning attributed to motherhood. Future research should examine how changes in MRIQ scores over time relate to trajectories of maternal well-being and parenting outcomes, and whether the instrument can be used to evaluate the effectiveness of programs designed to foster a more integrated, flexible, and compassionate experience of becoming a mother.

## Figures and Tables

**Table 1:** Sociodemographic characteristics of the EFA subsample (n = 83).

Variables	n	%	Mean (SD)
Age			34.70 (5.55)
Outcomes			
<13,737 € gross per year	16	19.28	
Between 13,737 € and 36,632 € gross per year	41	49.40	
>36,632 € gross per year	11	13.25	
Completed studies			
Primary Education	1	1.20	
Secondary Education	2	2.41	
Intermediate Vocational Training	8	9.64	
Advanced Vocational Training or Baccalaureate	23	27.71	
Bachelor’s Degree	19	22.89	
Master’s Degree	28	33.74	
Doctorate	2	2.41	
Currently working			
Yes	60	72.29	
No	23	27.71	
Home country			
Spain	76	91.57	
Others	7	8.43	
Perceived sleep quality			
Very Good	12	14.46	
Good	33	39.76	
Average	30	36.15	
Bad	6	7.23	
Very Bad	2	2.41	
Hours of sleep			7.51 (1.29)
4 h	2	2.41	
5 h	4	4.82	
6 h	8	9.64	
7 h	22	26.51	
8 h	36	43.37	
9 h	7	8.43	
10 h	3	3.61	
12 h	1	1.21	
Gestational age (weeks)			22.89 (9.83)
Time to conception (months)			7.34 (11.28)
Previous children			
Yes	50	60.24	
No	33	39.76	
Planned pregnancy			
Yes	65	78.31	
Partially	10	12.05	
No	8	9.64	
Pregnancy complications (no/yes)			
Yes	12	14.46	
No	71	85.54	
Previous abortion			
Yes	16	19.28	
No	21	25.30	

Note: N = 83.

**Table 2:** EFA rotated factor matrix: overview of 10-item and 8-item solutions (n = 83).

Item	10-Item Solution				8-Item Solution (After Removing Low-Loading or Cross-Loading Items)			
	F1 Loadings	F2 Loadings	R^2^	Complexity	F1 Loadings	F2 Loadings	R^2^	Complexity
1	**0.81**	0.07	0.72	1.02	0.13	**0.75**	0.70	1.06
2	**0.75**	−0.12	0.50	1.05	−0.20	**0.75**	0.44	1.14
3	**0.76**	−0.05	0.54	1.01	0.19	**0.49**	0.59	1.29
4	**0.80**	−0.03	0.61	1.00	0.00	**0.88**	0.78	1.00
5	0.12	**0.80**	0.75	1.05	**0.85**	−0.17	0.90	1.05
6	−0.13	**0.45**	0.17	1.17				
7	0.26	**0.61**	0.57	1.34	**0.66**	0.16	0.59	1.12
8	−0.08	**0.56**	0.58	1.04	**0.53**	−0.07	0.20	1.04
9	**0.73**	0.10	0.20	1.04				
10	0.17	**0.67**	0.58	1.13	**0.61**	0.14	0.48	1.10
	F1	F2			F1	F2		
r with F2	0.45	-			0.57	-		
Explained variance	32.05%	21.2%			28.4%	28.1%		

*Note*. F1–F2, factors one to two; R^2^, communalities; r, Pearson’s correlation coefficient; Loadings in bold indicate the strongest factor association for every item.

**Table 3:** Fit indices for measurement models.

Model	npar	$\chi^{2}$	*df*	CFI	TLI	NFI	RMSEA	SRMR	Model Comparison	${\Delta \chi }^{2}$	Δ*df*	*p*
M1. Bifactor model (General MRIQ factor and two specific dimensions of maternity identity, MI and BMR)	56	15.141	12	0.975	0.942	0.968	0.039 [0.000, 0.092]	0.033				
M2. Two-factor model (MI and BMR)	49	40.334	19	0.966	0.950	0.955	0.081 [0.046, 0.115]	0.057	M2-M1	24.463	7	0.005
M3. One-factor model (General dimension of model MRIQ)	48	263.249	20	0.817	0.743	0.808	0.266 [0.238, 0.295]	0.139	M3-M1	213.95	8	0.000

*Note*. MI = Maternal Identity, BMR = Balance of the maternal role.

**Table 4:** Group comparisons of MRIQ-P scores by prior motherhood and anxiety diagnosis (n = 173).

	No Anxiety Diagnosis (n = 123)		Anxiety Diagnosis (n = 50)				
	M	SD	M	SD	*U*	*p*	$r_{rb}$
MRIQ-P. MI ^a^	3.98	1.08	3.67	1.19	3592	0.041	−0.168
MRIQ-P. BMR ^a^	4.15	1.14	3.74	1.16	3686.5	0.020	−0.199
MRIQ-P. Total score ^a^	4.07	0.95	3.70	0.95	3736.5	0.013	−0.215

*Note*. M = mean, SD = Standard Deviation, *U* = Mann–Whitney statistic, *p* = *p*-value of the U-statistic; ^a^ = normality not assumed, $r_{rb}$ = rank-biserial correlation effect size, MI = Maternal Identity, BMR = Balance of the maternal role.

**Table 5:** Descriptive statistics, intercorrelations, Cronbach’s alphas, and bivariate correlations (r) for MRIQ-P scores across demographic, contextual, and psychological variables (n = 173).

	Scores Range	M	SD	MRIQ-P. MI	MRIQ-P. BMR	MRIQ-P. Total Score	α
MRIQ-P. MI	1–6	3.95	1.11	-	0.43 ***	0.84 ***	0.98
MRIQ-P. BMR	1–6	4.00	1.12	-	-	0.85 ***	0.98
MRIQ-P. Total score	1–6	3.98	0.96	-	-	-	0.96
Sociodemographic variables							
Age	22–45	34.44	4.73	−0.02	0.12	0.06	-
Income	1–3	2.03	0.70	−0.06	0.07	0	-
Educational level	1–7	5.06	1.09	−0.09	0.03	−0.04	-
Gestational age (weeks)	1–41	21.75	9.80	−0.09	−0.04	−0.07	-
Time to conception (months)	0–120	9.32	15.78	−0.04	0.02	−0.01	-
Hours of sleep	4–14	7.46	1.28	0.03	0.05	0.05	-
Perceived sleep quality	1–5	2.66	0.88	−0.10	−0.14	−0.14	-
Previous children (no/yes)	1–2	1.40	0.49	0.16 *	−0.14	0.01	-
Planned pregnancy (no/yes)	1–2	1.90	0.30	−0.06	0.02	−0.03	-
Pregnancy complications (no/yes)	1–2	1.18	0.38	0.03	0.06	0.05	-
Psychosocial variables							
Self-esteem	10–40	29.80	6.14	0.17 *	0.52 ***	0.41 ***	0.91
PANAS: Positive affect	10–50	32.62	7.19	0.31 ***	0.42 ***	0.43 ***	0.87
PANAS: Negative affect	10–49	24.58	8.97	−0.16 *	−0.43 ***	−0.35 ***	0.92
Maternal ambivalence: Doubts	6–24	12.39	4.31	−0.30 ***	−0.31 ***	−0.36 ***	0.88
Maternal ambivalence: Conviction	4–16	12.77	2.54	0.61 ***	0.41 ***	0.60 ***	0.82
Psychological wellbeing							
General Anxiety	1–4	2.29	0.64	−0.15 *	−0.44 ***	−0.36 ***	0.75
General Depression	1–4	1.68	0.60	−0.15 *	−0.52 ***	−0.40 ***	0.75
Prenatal distress	10–50	29.10	6.79	−0.13	−0.26 ***	−0.23 ***	0.68

*Note*. M = mean, SD = Standard Deviation, MI = Maternal Identity, BMR = Balance of the maternal role, α = Cronbach’s alpha. * *p* < 0.05. *** *p* < 0.001.

**Table 6:** Multiple linear regression examining the independent contributions of MRIQ-P dimensions to antenatal depression, dispositional guilt, and life satisfaction (n = 173).

Predictor Variables	Criterion Variables								
	Antenatal Depression			Dispositional Guilt			Life-Satisfaction		
	Step 1	Step 2	Step 3	Step 1	Step 2	Step 3	Step 1	Step 2	Step 3
Age	−0.090	−0.011	−0.002	−0.033	0.028	0.044	0.018	−0.048	−0.047
Gestational age	−0.162 *	−0.131 *	−0.139 *	−0.048	−0.017	−0.027	0.032	0.042	0.059
Pregnancy complications	−0.214 **	−0.177 **	−0.195 ***	−0.103	−0.077	−0.104	0.122	0.076	0.094
Perceived social support		−0.310 ***	−0.217 ***		−0.181 **	−0.041		0.530 ***	0.439
Cognitive Fusion		0.521 ***	0.487 ***		0.464 **	0.411 ***		−0.119	−0.093
MRIQ-P. MI			0.006			0.055			0.154 *
MRIQ-P. BMR			−0.179 *			−0.290 ***			0.110
R^2^	0.084	0.527	0.547	0.014	0.301	0.347	0.016	0.338	0.378
∆R^2^		0.443 ***	0.020 *		0.287 ***	0.046 **		0.322 ***	0.040 **

Note: All reported statistics are standardized coefficients (*β*). * *p* < 0.05. ** *p* < 0.01. *** *p* < 0.001.

**Table 7:** Measurement invariance of the MRIQ across primiparous and multiparous women (n = 173).

Model	χ^2^	df	CFI	TLI	NFI	RMSEA	SRMR	Difference	Δχ^2^	Δdf	p	ΔCFI	ΔTLI	ΔRMSEA	ΔSRMR
M1 Configural	29.543	24	0.991	0.979	0.956	0.052	0.034	*-*	*-*	*-*	*-*	*-*	*-*	*-*	*-*
M2 Metric	42.563	37	0.991	0.986	0.936	0.042	0.047	M2–M1	13.020	13	0.4463	0.000	0.007	−0.010	0.013
M3 Scalar	47.942	42	0.990	0.987	0.928	0.040	0.053	M3–M2	5.380	5	0.3713	−0.001	0.001	−0.001	0.006
M4 Strict	57.267	50	0.988	0.987	0.914	0.041	0.062	M4–M3	9.324	8	0.3157	−0.002	0.000	0.001	0.01

## Data Availability

The data presented in this study are available upon request from the corresponding author. The data are not publicly available due to privacy restrictions.
